# The complete mitochondrial genome of the Xinyuan honey bee, *Apis mellifera sinisxinyuan* (Insecta: Hymenoptera: Apidae)

**DOI:** 10.1080/23802359.2019.1705927

**Published:** 2020-01-10

**Authors:** Jialin Yang, Ziwei Jiao, Xuemei Wen, Bei Liu, Jiaxing Huang, Guiling Ding

**Affiliations:** a Yili Prefecture Agricultural and Rural Bureau, Xinjiang, China;; b College of Bio-and Geo-Sciences, YiLi Normal University, Xinjiang, China;; c Key Laboratory of Pollinating Insect Biology of the Ministry of Agriculture and Rural Affairs, Institute of Apicultural Research, Chinese Academy of Agricultural Sciences, Beijing, China

**Keywords:** Mitochondrial DNA, single molecule real-time sequencing, *Apis mellifera sinisxinyuan*

## Abstract

We analyzed the complete mitochondrial genome of the recently discovered Xinyuan honey bee, *Apis mellifera sinisxinyuan* using single molecule real-time sequencing. The mitochondrial genome of *A. m. sinisxinyuan* is a circular molecule of 16,886 bp, comprising 13 protein-coding genes, 22 tRNA genes, 2 rRNA genes and a control region rich in A + T. Phylogenetic analysis using 13 protein-coding genes supports a close relationship to another M-lineage honey bee, *A. m. mellifera*.

*Apis mellifera* has a wide distribution in Europe, western Asia and Africa. There are at least 27 morphologically and geographically distinct subspecies divided into five lineages (A, Y, M, C and O lineage) (Ruttner 1988; Sheppard and Meixner 2003; Chen et al. 2016; Dogantzis and Zayed 2019). The newly discovered *A. mellifera* subspecies *Apis mellifera sinisxinyuan*, was clustered in the M-lineage and postulated to diverge from *A. m. mellifera* about 132 KYA. *A. m. sinisxinyuan* is winter-tolerant and adapted to temperate climates (Chen et al. 2016). Currently, *A. m. sinisxinyuan* is found only in limited areas and little information is available about its basic knowledge (Chen et al. 2016; Zhao et al. 2018). Here, we report the complete mitochondrial genome of *A. m. sinisxinyuan* (GenBank: MN733955) sequenced from adult workers of one colony. This study would be helpful to better elucidate the evolution and adaptation of the honey bee.

Adult workers were collected from Xinyuan prefecture (43.2533°N, 83.8043°E) and stored in the Institute of Apicultural Research, Chinese Academy of Agricultural Sciences (accession number: 2019-0025). Genomic DNA was extracted from the thoracic muscle and single molecule real-time (SMRT) sequencing (PacBio RSII, Menlo Park, CA) was performed following standard procedures. The sequenced reads were assembled using Canu 1.8 (Koren et al. 2017). Protein coding genes (PCGs), transfer RNA (tRNA) and ribosomal RNA (rRNA) genes were identified with MITOS WebServer (Bernt et al. 2013) and confirmed by comparison with the reference sequences deposited in the GenBank database. We used MAFFT v7.271 (Katoh and Standley 2013) to align the 13 PCGs of 22 species and subspecies. The phylogenetic tree was constructed using MrBayes v3.2.5 (Ronquist et al. 2012) with the GTR + I + G model selected by jModelTest v2.1.7 (Darriba et al. 2012).

The mitochondrial genome of *A. m. sinisxinyuan* is a closed loop of 16,886 bp, with 43.5% A, 41.7% T, 9.4% C, and 5.4% G. It consists of 13 PCGs, 22 tRNA genes, two rRNA genes, and one putative control region rich in A and T. Nine PCGs and 14 tRNA genes are encoded on the heavy strand, while the remaining four PCGs, 8 tRNAs and 2 rRNAs on the light strand. The *ATP8* and *ATP6* genes share 19 nucleotides. The start codon is ATT for six PCGs; ATG for *ATP6*, *COIII* and *Cytb*; ATA for *COI*, *ND3* and *ND4* and ATC for *ND6*. All PCGs end with a TAA stop codon. The 12S rRNA and 16S rRNA are 785 bp (81.5% AT) and 1337 bp (84.6% AT), respectively. The 22 tRNAs vary from 63 bp (tRNA-Ser and tRNA-Gln) to 78 bp (tRNA-Thr), and all fold into cloverleaf secondary structures.

The phylogenetic tree indicates that *A. m. sinisxinyuan* clusters with *A. m. mellifera* (Figure 1). This is consistent with the result reported in previous study, which predicted a sister relationship between *A. m. mellifera* and *A. m. sinisxinyuan* using polymorphic SNPs in whole genome and the mitochondrial DNA fragment (tRNA ILE and part of the *ND*2 gene) (Chen et al. 2016).

**Figure 1.**
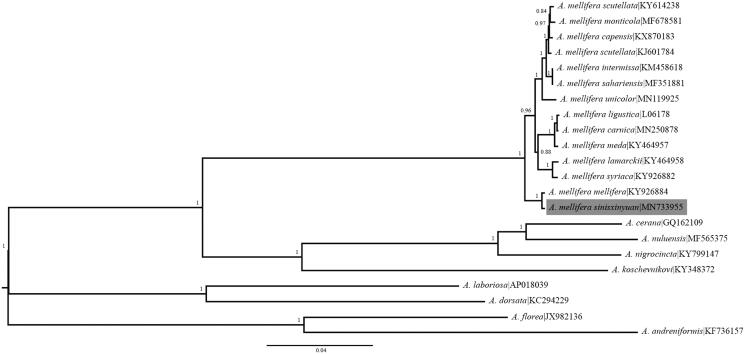
Phylogenetic tree showing the relationship between *Apis mellifera sinisxinyuan* and 21 other *Apis* species and subspecies. Values at each node are Bayesian posterior probabilities for the groups. The *scale bar* represents 0.04 substituions per nucleotide site. The GenBank accession numbers are listed after the species and subspecies names.
